# A new, conspicuously pigmented *Pyrrhulina* (Teleostei: Characiformes: Lebiasinidae) from the Río Tigre, Loreto, Peru

**DOI:** 10.1111/jfb.70371

**Published:** 2026-02-27

**Authors:** Andre Netto‐Ferreira, Lorena Vieira, Taina Souza, Morgan Ruiz‐Tafur, James Garcia‐Ayala

**Affiliations:** ^1^ Laboratório de Ictiologia, Departamento de Zoologia, Instituto de Biociências Universidade Federal do Rio Grande do Sul Porto Alegre Brazil; ^2^ Laboratório de Citogenética e Genética Animal, Programa de Pós‐graduação em Ecologia e Conservação da Biodiversidade Departamento de Biologia e Zoologia – IB Universidade Federal de Mato Grosso Cuiabá Brazil; ^3^ Laboratorio de Taxonomía de Peces Instituto de Investigaciones de la Amazonía Peruana (IIAP). San Juan Bautista Peru; ^4^ Laboratório de Biologia e Genética de Peixes, Departamento de Biologia Estrutural e Funcional, Setor de Morfologia Universidade Paulista ‘Júlio de Mesquita Filho’, Instituto de Biociências de Botucatu – UNESP Botucatu Brazil

**Keywords:** integrative taxonomy, Neotropics, *Pyrrhulina spilota*, *Pyrrhulina vittata*, systematics

## Abstract

A new species of *Pyrrhulina* is described based on morphological and molecular evidence. *Pyrrhulina punctata* is distinguished from all congeners by the presence of a series of 7 to 16 irregular blotches of dark pigmentation on the flanks, equally marked in juveniles and adult specimens, the presence of 26–28 lateral‐line scales, 17–21 maxillary teeth, 23–25 outer premaxillary teeth, 40–41 inner premaxillary teeth and 23 outer dentary teeth, the distinctly elongate rays on dorsal, pelvic and anal fin on adult males, the presence of irregular dark marks on the basal portion of anal‐fin rays and intervening membranes, and the presence of a second dark blotch on the distal tip of longest dorsal‐fin rays. The molecular data indicate a closer relationship with *Pyrrhulina spilota*, corroborating the morphological evidence and the shared colouration pattern. Besides the diagnostic characters allowing the recognition of both species, their distinction was corroborated by all species delimitation algorithms [Assemble Species by Automatic Partitioning (ASAP), Poisson Tree Processes (PTP) and Generalized Mixed Yule Coalescence (GMYC)] employed herein. In addition, the occurrence of *P. spilota* in the Madeira basin is refuted as the registers were based on misidentifications of *Pyrrhulina vittata.*

## 1 INTRODUCTION

The genus *Pyrrhulina* Valenciennes currently houses 19 valid species (Fricke et al., 2023). Most of its representatives lack remarkable pigmentation on the flanks besides an overall silver colouration followed by three to five longitudinal rows of blotches ranging from yellow to red (Netto‐Ferreira & Marinho, 2013; Vieira & Netto‐Ferreira, 2019; Weitzman & Weitzma, 2003; Zarske & Géry, 1997). Exceptions to the pale colouration can be summarized in three distinct patterns (Vieira & Netto‐Ferreira, 2019). The first consists of the presence of a irregularly arranged dark pigmentation on the flanks, as presented by *Pyrrhulina brevis* Steindachner, *Pyrrhulina lugubris* Eigenmann and *Pyrrhulina obermulleri* Eigenmann; the second consists of a longitudinal stripe with variable extent in *Pyrrhulina beni* Myers, *Pyrrhulina marilynae* Netto‐Ferreira and Marinho, *Pyrrhulina semifasciata* Steindachner and *Pyrrhulina zigzag* Zarske and Géry; and the third, in which a series of conspicuous, large dark blotches are present on the flanks, shared by *Pyrrhulina spilota* Weitzman and *Pyrrhulina vittata* Regan.

In recent years, studies focusing on cytogenetic data of *Pyrrhulina* have been frequent (de Moraes et al., 2017, 2019, 2021, 2023), with molecular research aimed at understanding the diversity in *Pyrrhulina* being less abundant. Ferreira et al. (2022) and Souza et al. (2023) were the first authors to use DNA data to evaluate species diversity and relationships within the genus. The present contribution employs the molecular information available to *Pyrrhulina* species to present the first integrative taxonomic study for the genus, aiming to describe a new species from the Río Tigre in Peru.

Despite its resemblance with *P. spilota*, the species has been recognized as a distinct species (Weitzman, personal communication; Romayna Rios, 2020) based on distinctive expression of the colouration of adults. The species has been known in the aquarium trade since at least 2001, when the first specimens were sent from Peru to Marilyn Weitzman by Julio Melgar as documented in M. Weitzman's (personal communication) notes: ‘In January 2001 I got a message that Julio Melgar had what appeared to be a new *Pyrrhulina* with more spots than *Pyrrhulina spilota* but from the same region and he offered to let me have some to examine. It is indeed a new species. It has a black stripe along the midside of the body that is broken into spots. The number of spots varies with the individual and even on each side of a fish. I was fortunate to have been on a Margarita Tours trip last July and at the village of Santa Ana upstream of Iquitos some *Pyrrhulina spilota* were brought for trade to our boat by some of the boys. There were many fine large specimens and I tried to get some home alive for observation but a long delay in Lima was too stressful for the larger ones, only two smaller ones survived. Stanley Weitzman described *Pyrrhulina spilota* in 1960 from aquarium specimens said to have come from the Iquitos region of Peru…’. In addition, the putative relationships of the species among its congeners are discussed, and the occurrence of *P. spilota* in the Madeira basin is evaluated.

## 2 MATERIALS AND METHODS

### 2.1 Morphological analyses

No specimen was examined in vivo, collected or killed during the present study. All specimens were previously catalogued in ichthyological collections. Counts and measurements follow those proposed by Fink and Weitzman (1974) and Netto‐Ferreira et al. (2011). All measurements were made point‐to‐point using callipers with 0.1 mm precision. Standard length (SL) is presented in millimetre, and all other measurements are presented as proportions of SL, except for head subunits, which are presented as proportions of head length (HL). Meristic data are given in the description, followed by the frequency of each count in parentheses and an asterisk indicating values for the holotype. Observations of teeth, vertebrae, supraneurals and procurrent caudal‐fin ray counts were obtained from cleared and stained (c&s) paratypes according to Taylor and Van Dyke (1985). Counts were made from the left side of specimens whenever possible. Vertebrae of the Weberian apparatus were considered four precaudal elements; transitional vertebrae (sensu Weitzman, 1962) were included in the count of precaudal vertebrae; and the fused PU1 + U1 of the caudal region was counted as a single element (Netto‐Ferreira et al., 2011). Patterns of circuli and radii were observed on scales sampled from the region between the lateral‐line series and the dorsal‐fin origin. Colour pattern nomenclature follows Weitzman (1966). Institutional abbreviations follow Sabaj (2020, 2023), with the addition of CIIAP: Colección Ictiológica del Instituto de Investigaciones de la Amazonía Peruana.

### 2.2 Molecular analyses and species delimitation

Genomic DNA from newly sequenced specimens was extracted from muscle or fin tissues preserved in 95% ethanol using the Wizard Genomic DNA Purification kit (Promega). We amplified the mitochondrial protein‐coding gene cytochrome c oxidase subunit I (COI) using the primers FishF1 and Fish2 (Ward et al., 2005). Polymerase chain reaction (PCR) reactions used a volume of 12.5 μL with 9.0 μL of double‐distilled water, 1.25 μL 5X buffer, 0.50 μL MgCl_2_ (50 mM), 0.50 μL dNTP mix, 0.25 μL of each primer at 10 μM, 0.20 μL Taq polymerase enzyme and 1.0 μL genomic DNA (10–50 ng). The PCR programme consisted of an initial denaturation (3 min at 95°C) followed by 35 cycles of chain denaturation (30 s at 94°C), primer hybridization (30–60 s at 52°C) and nucleotide extension (60 s at 72°C). PCR products were seen on a 1% agarose gel and employed with dye terminators for the sequencing procedure (BigDye Terminator version 3.1 Cycle Sequencing Ready Reaction Kit, Applied Biosystems). Samples were placed into an ABI 3130‐Genetic Analyser automated sequencer (Applied Biosystems) at the Instituto de Biociências da Universidade Estadual Paulista, Botucatu, Brazil.

Sequences from two individuals of the species described here, and six *Pyrrhulina* species available on BOLD Systems, were utilized in species delimitation analyses (Table 1). Raw sequences were edited using Geneious 7.1.3 software (Kearse et al., 2012). The final alignment, detection of stop codons, pseudogenes, deletions, insertions and the calculation of intra‐ and interspecific genetic distances were performed using Mega version 11 software (Tamura et al., 2021). Data quality was assessed by applying the Nucleotide Saturation Test in DAMBE version 7 (Xia et al., 2003). For the validation of the new species, we applied three types of species delimitation methods detailed below. The Assemble Species by Automatic Partitioning software (ASAP, Puillandre et al., 2021) is based on genetic distance using the K2P substitution model, estimated on the site https://bioinfo.mnhn.fr/abi/public/asap/asapweb.html, with all other parameters set to default values. For coalescence‐based methods, we estimated the best nucleotide evolution model using JModeltest2 version 2.1.6, implemented on the CIPRES platform available at https://www.phylo.org/portal2 (Miller et al., 2010). Poisson Tree Processes (PTP) analysis involved estimating groups from the maximum likelihood tree of IQ‐TREE (Nguyen et al., 2015). In this analysis, two sequences of *Copella arnoldi* Regan, 1912 (BOLD accession numbers GBOL2183‐17 and GBOL2184‐17) were used as out‐groups. This non‐ultrametric tree served as input for PTP on the site https://species.h-its.org/ (Zhang et al., 2013), with the parameters rooted, 40,000 Markov chain Monte‐Carlo (MCMC) generations, analysis without considering the out‐group. All other configurations were set to default values. In Bayesian Evolutionary Analysis Sampling Trees (BEAST2, Bouckaert et al., 2014), an ultrametric tree was constructed, a prerequisite for the Generalized Mixed Yule Coalescence (GMYC) method, with the following parameters: HKY + G model, gamma shape, relaxed molecular clock with log‐normal distribution and Yule speciation model. Three independent runs were conducted, each with 30 million generations, based on the MCMC method. We confirmed using Tracer version 1.6 that the logs from the three runs were sufficient to verify Effective Sample Size (ESS >200). After this step, we combined the trees with a 25% burn‐in using Treeannotator version 1.8 (version available at CIPRES). To perform the GMYC analysis, we input the ultrametric tree from BEAST2 into the R version 3.6.3 programme, running the analysis with a single threshold parameter using the SPLITs package (SPecies LImits by Threshold Statistics; Monaghan et al., 2009).

**TABLE 1:** Tissue sample, locality and accession numbers of each sequence obtained from the BOLD Systems for the species of *Pyrrhulina* compared in the present study.

*Pyrrhulina* species	Locality	Co‐ordinate	Bold systems/GenBank accession number
*P. punctata*	Playa Mabumdi, Peru/Río Tigre basin	03°14′42″S, 74°59′22″W	PV719272 PV719273
*P*. *aff. australis*	Pontes e Lacerda, Brazil/Guaporé basin	15°21′30.24″S, 59°15′10.56″W	PYR001‐22PYR002‐22
*P. spilota*	Puchana, Peru/Mómon River, Amazon basin	03°41′24.00″S, 73°16′12.00″W	GBMND24650‐21GBMND24651‐21
*P. obermulleri*	Porto Velho, Brazil/Madeira basin	09°02′36.31″S, 64°36′34.99″W	PYR020‐22PYR021‐22
*P. australis*	Poconé, Brazil/Bento Gomes River, Paraguay basin	16°09′07.39″S, 56°37′02.85″W	PYR033‐22PYR034‐22
*P. marilynae*	Sorriso, Brazil/Teles Pires River, Tapajós basin	12°57′17.68″S, 55°44′37.82″W	PYR023‐22
*P. filamentosa*	Sipaliwini, Suriname/Paloemeu River, Maroni basin	03°10′41.99″N, 55°25′09.12″W	GBOL1032‐16GBOL1033‐16

## 3 RESULTS

*Pyrrhulina punctata*, new species.

urn:lsid:zoobank.org:act:D4D389BC‐C6B5‐40F0‐A058‐3BD4A8F836D1.

*Pyrrhulina* sp. ‘mojarrita:’

Romayna Rios, 2020: 58 (Figure).

**Holotype. CIIAP 3919**, 1, 51.8 mm SL, Peru, Loreto, Quebrada Brashico, Río Tigre, 03°12′08.1″S 75°00′39.1″W, 08 August 2019, C. R. Romaina‐Rios.

**Paratypes**. All from Peru, from the Río Tigre basin: CIIAP 605, 7, 19.6–49.3 mm SL, UFRGS 30593, 2, 50.2–51.2 mm SL; 2 c&s 43.8–48.3 mm SL, same data as holotype. CIIAP 606, 19, 17.2–58.6 mm SL, Playa Mabumdi, 03°14′42″S, 74°59′22″W, 08 August 2019, C. R. Romaina‐Rios; CIIAP 607, 5, 22.5–43.7 mm SL, INPA‐ICT 060700, 2, 38.8–49.3 mm SL; LBP 35052, 3, 34.3–46.0 mm SL; MZUSP 129873, 2, 33.6–41.0 mm SL, Quebrada Monterriguillo, 03°11′7.6″S, 75°01′37.8″W, 18 August 2019, C. R. Romaina‐Rios; CIIAP 608, 6, 33.1–56.2 mm SL, Quebrada Boca Aleman, 02°43′13.1″S, 75°03′55.5″W, 01 August 2019, C. R. Romaina‐Rios.

**Diagnosis**. *P. punctata* sp. n. is promptly distinguished from all congeners, except *P. spilota* and *P. vittata*, by the presence of a series of irregular blotches of dark pigmentation on the flanks (vs. dark pigmentation on the flanks lacking a distinct pattern or represented only by the primary stripe and/or an anterior round blotch in male specimens in *Pyrrhulina australis*, *P. brevis*, *Pyrrhulina capim*, *Pyrrhulina eleanorae*, *Pyrrhulina elongata*, *Pyrrhulina filamentosa*, *Pyrrhulina laeta*, *P. semifasciata* and *P. stoli*; flanks conspicuously pigmented on adult male specimens in *P. lugubris* and *P. obermulleri*; and the presence of a conspicuous dark stripe on *P. beni*, *P. marilynae* and *P. zigzag*). The new species can be distinguished from *P. spilota* and *P. vittata* by the presence of a higher number of narrow, usually one scale wide, blotches throughout the flanks (from 7 to 16 vs. 2–5 on *P. spilota* and *P. vittata*). *P. punctata* sp. N. can be further distinguished from *P. vittata* by the presence of 26–28 lateral‐line scales (vs. 20–23), the higher number of teeth (17–21 maxillary teeth, 23–25 outer premaxillary teeth, 40–41 inner premaxillary teeth and 23 outer dentary teeth vs. 1–11 maxillary, 9–16 outer premaxillary, 20–30 inner pre maxillary and 10–13 outer premaxillary), the distinctly elongate rays on dorsal, pelvic and anal fins on adult males (vs. fins lacking distinctly elongate rays), the presence of dark marks on the posteriormost anal‐fin rays and intervening membranes (vs. dark marking, usually absent or distributed along the anal‐fin margin). The new species can be further distinguished from *P. spilota* by the blotches of adult specimens being as conspicuous as those of juveniles, and the presence of a second dark blotch on the distal tip of longest dorsal‐fin rays (vs. blotches on flanks of adults inconspicuously marked, dorsal‐fin rays of male specimens with single blotch onto median portion of rays – see Remarks).

### 3.1 Description

**FIGURE 1:**
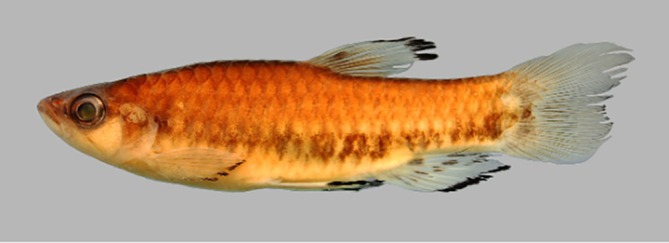
*Pyrrhulina punctata*, new species, CIIAP 3919, holotype, 51.8 mm standard length (SL), Peru, Loreto, Quebrada Brashico, Río Tigre, Río Marañón basin.

Morphometric data of the holotype and 17 paratypes of *P. punctata* are presented in Table 2. Lateral view of male holotype and female paratype is presented in Figure 1. Body cylindrical, slightly elongate. Greatest body depth anterior to dorsal‐fin origin, between pectoral and pelvic fins. Dorsal profile of head straight, slightly concave from upper lip to anterior scales covering parietal and supraoccipital. Dorsal profile of body convex from that point to dorsal‐fin base, becoming straight from that point to origin of anterodorsal procurrent ray of caudal fin. Ventral profile of head and trunk convex from lower lip to pelvic‐fin origin, becoming slightly straight from that point to anal‐fin origin. Ventral profile of anal‐fin base gently concave, caudal peduncle straight from that point to origin of anteroventral procurrent ray of caudal fin.

**TABLE 2:** Morphometric data of *Pyrrhulina punctata*.

Counts	Holotype	*n*	Males			Females and juveniles		
			Range	Mean	SD	Range	Mean	SD
Standard length	51.8	17	33.6–51.8	45.2		22.2–42.0	30.0	
Depth of dorsal fin	28.2	17	26.7–30.2	28.1	0.9	26.4–28.4	27.5	0.8
Snout to anal‐fin origin	76.7	17	76.3–79.9	7.8	1.0	75.3–78.3	76.6	1.1
Snout to pelvic‐fin origin	54.1	17	51.6–54.8	53.0	1.0	49.9–53.5	51.6	1.3
Snout to dorsal‐fin origin	59.4	17	58.7–62.2	60.0	1.2	58.0–62.4	59.6	1.4
Dorsal‐fin origin to caudal base	42.8	17	40.8–44.0	42.8	1.1	40.0–45.2	43.2	1.6
Dorsal‐fin length	31.8	17	25.8–33.5	29.9	2.3	27.1–29.5	28.2	0.8
Caudal peduncle length	16.1	17	14.9–18.4	16.4	1.0	16.0–18.7	17.3	0.9
Caudal peduncle depth	16.7	17	14.6–17.8	15.8	0.9	12.5–15.4	14.1	0.8
Anal‐fin length	32,5	17	26.2–32.9	28.9	2.5	19.6–26.3	22.8	2.4
Anal‐fin base	9.6	17	9.6–11.5	10.6	0.6	8.0–10.4	9.7	0.8
Pelvic to anal‐fin origin	24.4	17	24.4–27.2	25.8	0.9	23.6–27.4	25.7	1.2
Pelvic‐fin length	27.2	17	21.2–27.2	24.3	1.8	19.0–22.5	21.0	1.2
Pectoral‐ to pelvic‐fin origin	27.0	17	22.2–28.2	26.6	1.8	24.9–27.5	25.9	0.9
Pectoral‐fin length	23.4	17	21.4–24.0	23.0	0.6	19.7–23.5	22.3	1.4
Snout to pectoral‐fin origin	27.4	17	24.0–27.4	25.8	0.9	25.3–28.5	26.9	1.2
Head length	27.8	17	25.7–28.3	27.0	0.8	26.9–28.9	28	0.7
Horizontal eye diameter	29.4	17	29.4–38.2	31.9	2.5	32.5–39.7	37.3	2.6
Distance snout tip to eye	31.2	17	28.5–31.4	30.0	1.1	24.5–31.1	26.7	2.1
Interorbital distance	36.3	17	35.0–38.0	36.4	0.9	34.0–38.0	36.7	1.2
Lower‐jaw length	43.7	17	39.3–44.4	42.2	1.8	40.6–44.9	42.5	1.4
Upper‐jaw length	39.1	17	36.1–40.0	38.1	1.1	32.9–38.8	36.2	1.8

*Note*: Values for the holotype included in the range of male specimens. *N* = 17.

Mouth superior. Premaxillary with two series of conical teeth; outer series with 19(1) or 23(1) teeth; inner series with 36(1) or 48(1) teeth. Maxillary with 14(1) or 18(1) conical teeth, anterior teeth largest in both sexes. Dentary with two series of conical teeth; outer series with 18(1) or 19(1); inner series with 52(1) or 61(1). Inner series of teeth gradually decreasing in size from symphysis to near coronoid process. Lower jaw protruding slightly beyond premaxillary. Branchiostegal rays 3(2); two articulating with anterior ceratohyal and one with posterior.

Scales cycloid, circuli restricted to anterior border, few radii converging and strongly anastomosed at focus, not forming cells. Lateral‐line series with 26(5), 27*(12) or 28(3) scales; none perforated. Longitudinal series of scales between dorsal and pelvic fins 7. Predorsal scales 10(3), 11*(14) or 12(3). First paired longitudinal series with 11(1), 12(11) or 13*(8) scales. Circumpeduncular scales 10*(19) or 11(1).

Pectoral‐fin rays i,10(5), i,11*(14) or i,12(1). Tip of longest pectoral‐fin ray anterior of vertical through pelvic‐fin origin. Pelvic‐fin rays i,7*(20); tip of longest pelvic‐fin ray reaching anal‐fin origin in adult males, but not in juveniles or females. Supraneurals 6(2), positioned anterior to neural spines of centra 6 to 12. First dorsal‐fin pterygiophore inserted posterior to neural spine of centrum 11(1) or 12(1). Dorsal‐fin rays ii,8*(20). Distal margin of extended dorsal fin somewhat rounded in females and lanceolate in adult males. Dorsal‐fin origin located distinctly closer to caudal fin than to snout tip. Base of last dorsal‐fin ray located before the vertical through anal‐fin origin. Anal‐fin rays iii,8*(20), with last ray adnate. Profile of extended anal fin slightly rounded in females, somewhat elliptical or lanceolate in males. First anal‐fin pterygiophore inserted posterior to haemal arch of centrum 18(1) or 19(1). Adipose fin absent. Caudal fin forked, upper lobe distinctly longer than lower. Caudal‐fin principal rays i,9/7,i(3), i,9/8,i*(15), i,9/10,i(1) or ii,10/7,i(1). Dorsal caudal‐fin procurrent rays 4(2). Ventral caudal‐fin procurrent rays 5(2). Precaudal vertebrae 18(2); caudal vertebrae 17(2).

### 3.2 Colour in alcohol

**FIGURE 2:**
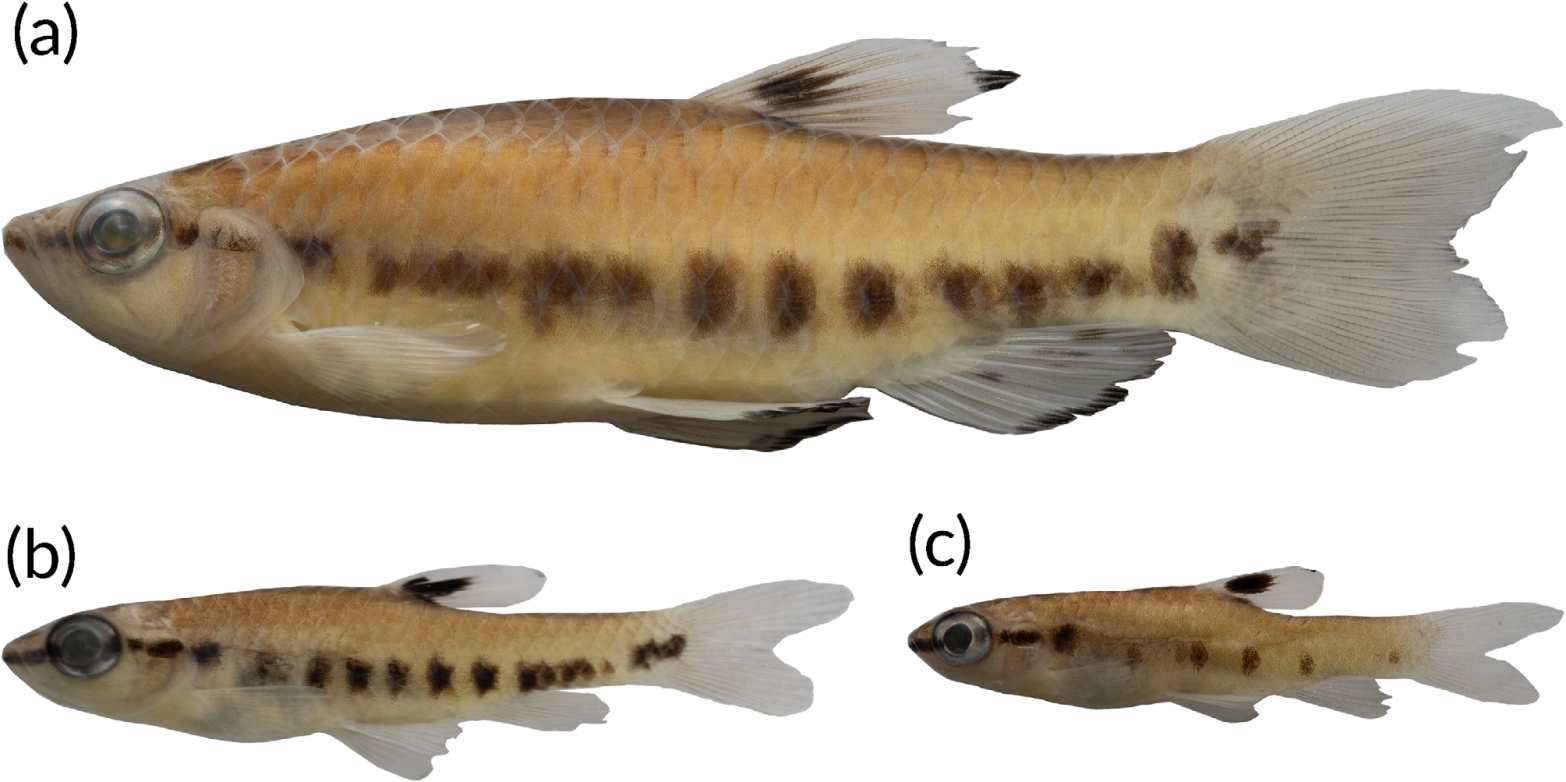
Variation in the colour pattern of *Pyrrhulina punctata* in lateral view showing (a) an adult male specimen [CIIAP 606, 44.86 mm standard length (SL)], (b) a larger juvenile (CIIAP 605, 19.77 mm SL), (c) a smaller juvenile (CIIAP 606, 16.44 mm SL).

Background colour yellowish. Dorsal portion of head light brown from the upper lip to scales overlying parietal bone. Lateral surfaces of head distinctly lighter than dorsum, with dark pigmentation becoming abruptly scarce ventral to primary stripe. Primary stripe heavily pigmented extending from the head, pigmenting both jaws, antorbital, infraorbitals 1 and 5 and eye, and reaching the pectoral girdle in the humeral area posteriorly. Ventral portion of head with few scattered chromatophores. Trunk pigmentation slightly counter shaded, with predorsal series strongly pigmented and longitudinal series of scales 1–3 with discrete brown pigmentation, becoming lighter ventrally; background colour of scales on series 4–6 distinctly lighter, except for those forming series of dark blotches. Mid‐lower portion of flanks with a series of irregular dark blotches, extending from pectoral girdle to median caudal‐fin rays, onto scales of series 4–6. Blotches variable in number and size, ranging from 7 to 16, usually one scale wide. Lowest number of blotches observed in juveniles below 28.4 mm SL; blotches increasing in number and width from 34.5 mm SL, and coalescing near 40.31 mm SL, forming irregular band of pigmentation on mid‐ventral portion of flanks (Figure 2). Abdominal region yellowish with scarce chromatophores, from isthmus to anal‐fin origin. Pectoral fin and pelvic mostly hyaline, except for pigmented distal margin in adult males (see Sexual dimorphism). Dorsal fin with round blotch at proximal portion of anteriormost rays and distinct dark pigmentation along the distal margin of all rays. Anal fin mostly hyaline, except for small, variably conspicuous dark blotches onto basal portion of first and seventh/eight branched rays, on distal margin of eighth branched, and distal margin of anal fin on male specimens (see sexual dimorphism), becoming more intensely marked during growth. Caudal fin mostly hyaline with scarce chromatophores, except for variably shaped blotch onto basal portion of median caudal‐fin rays.

### 3.3 Sexual dimorphism

Adult males of *P. punctata* have the common sexual dimorphism of Lebiasinidae (Netto‐Ferreira et al., 2011; Netto‐Ferreira & Marinho, 2013), in which the anal‐fin rays and all intervening membranes are distinctly thicker and longer than in females, ultimately resulting in broader anal fins in male specimens (Marinho & Menezes, 2017). Adult males have dark pigmentation at the distal margin of the pectoral, dorsal anal and pelvic fins forming a distinct dark band on each fin. Pelvic, dorsal and anal fins of males distinctly longer than those of females.

### 3.4 Distribution

**FIGURE 3:**
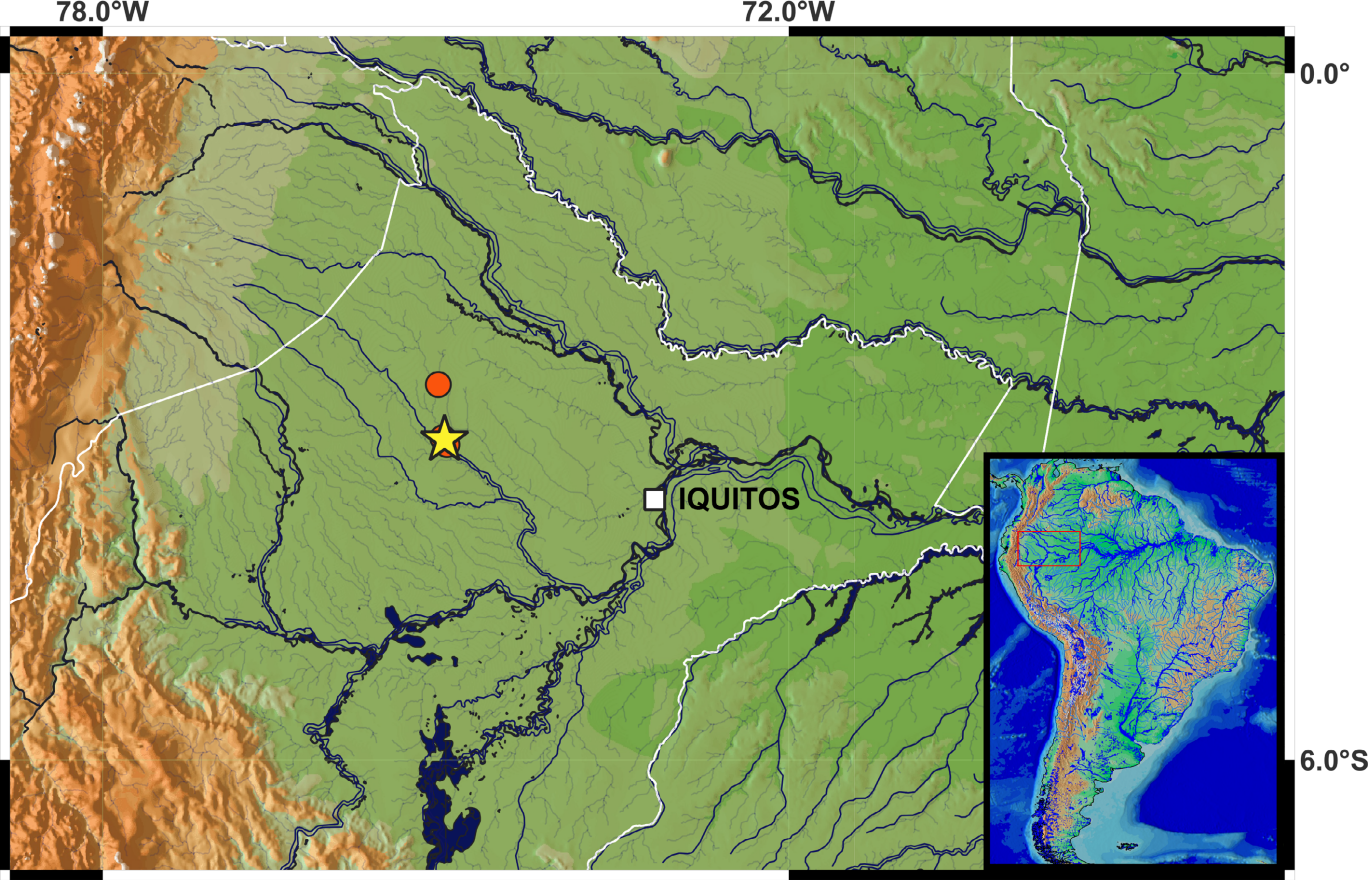
Map of northern South America showing the type locality (yellow star) and collections sites (red circles) of *Pyrrhulina punctata* and adjoining area. Some symbols represent more than one collection site.

*P. punctata* sp. n. is currently known only from the Río Tigre and its tributaries, itself a tributary of the Río Marañón in Peru (Figure 3).

**Etymology:** The specific epithet derives from the Latin *punctata*, meaning spotted or dotted, in allusion to the series of dark blotches on the flanks and fins of its representatives.

**Conservation status:** *P. punctata* occurs in well‐preserved tropical forest areas characterized by the presence of small stream, sandy bottoms and slow current speeds. Despite not being frequent or abundant, all collection sites are within the boundaries of the Protected Natural Area (ANP) Pucacuro National Reserve (RNPU), an area considered important for the conservation of biological diversity due to its exceptional species richness and endemism (SERNANP, 2013). Therefore, we suggest that *P. punctata* be classified as least concern (LC) according to the International Union for Conservation of Nature (IUCN) categories and criteria (IUCN 2026).

### 3.5 Species delimitation

**FIGURE 4:**
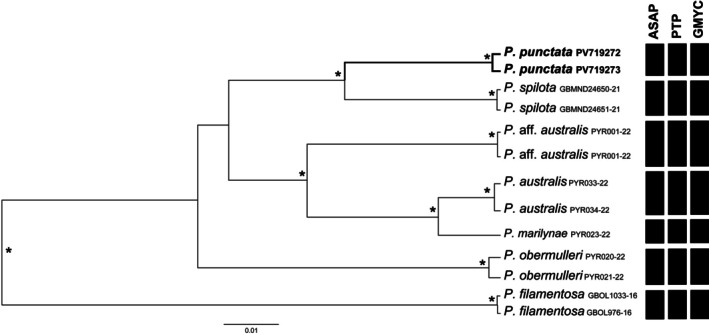
Bayesian tree and species delimitation recognized using the Assemble Species by Automatic Partitioning (ASAP), Poisson Tree Processes (PTP) and Generalized Mixed Yule Coalescence (GMYC) methods. Asterisks indicate monophyletic species.

The size of the final alignment of 13 partial sequences of the COI gene from *Pyrrhulina* species was 627 base pairs (bp), of which 125 bp were parsimoniously informative for the analyses. The presence of stop codons, pseudogenes, deletions and insertions was not observed in the final matrix. The dataset did not show saturation; the Iss values (*R* ^2^ = 0.1974) were lower than Iss.c (*R* ^2^ = 0.7352). The means of inter‐ and intraspecific distances for *Pyrrhulina* species can be seen in Table 3. The lowest interspecific distance between *P. punctata* and other species in the group, particularly *P. spilota*, in this dataset had the smallest genetic distance in relation to *P*. *punctata* (5.40%), whereas the genetically most distant species from *P*. *punctata* was *P. filamentosa* (13.79%). The results from the three delimitation methods (ASAP, PTP and GMYC) employed in this study were congruent in species delimitation and support the recognition of *P. punctata* as a distinct species and can be observed in Figure 4. Clades according to the Bayesian tree showed high posterior probability in all branches (> 95%), forming monophyletic groups [(*P. punctata, P. spilota*) *P*. aff. *australis*, *P. australis*, *P. marilynae*], *P. obermulleri* and *P. filamentosa*.

**TABLE 3:** Mean K2P genetic distances of *Pyrrhulina* species analysed in this study (%).

Species	1	2	3	4	5	6	7
1. *Pyrrhulina punctata*	**0**						
2. *Pyrrhulina* aff. *australis*	8.31	**0**					
3. *Pyrrhulina spilota*	5.4	8.3	**0**				
4. *Pyrrhulina obermulleri*	8.19	8.02	9.09	**0**			
5. *Pyrrhulina australis*	8.61	6.88	9.59	8.87	**0**		
6. *Pyrrhulina marilynae*	7.72	5.35	8.53	8.78	2.03	**0**	
7. *Pyrrhulina filamentosa*	13.79	12.96	15.01	14.39	11.28	10.41	**0**

*Note*: Interspecific distances below diagonal. Bold numbers represent intraspecific genetic distances.

### 3.6 Remarks

*Pyrrhulina punctata* shares with all congeners the diagnostic characters defining the genus, as suggested by Géry (1977): the presence of two rows of teeth in the premaxilla, the opercular membrane united to the isthmus ventrally and the absence of the postcleithrum 3. Despite the low taxon and lineage sampling, the sister‐group relationship between *P. punctata* and *P. spilota* indicates a common origin of the pigmentation pattern shared by the new species and the latter. Although specimens of *P. vittata* were not included in the present analyses, it is likely that the pigmentation pattern of the latter is not homologous to that of the pair *P. spilota* + *P. punctata*, as that species seems to be more closely related to the *P. australis* species group as suggested by Netto‐Ferreira and Marinho (2013) based on the reduced number of precaudal vertebrae; the comparatively shorter dorsal‐, anal‐ and caudal‐fin rays in males; and the reduction or loss of the postcleithrum 2. In addition to the anatomical distinction of *P. vittata* from *P*. *punctata* + *P. spilota*, the number and position of the blotches seem to vary considerably between the species, further indicating independent origins of a similar pattern. In *P. spilota*, usually four of five well‐marked dark blotches are usually present, with the anteriormost lying adjacent to the primary stripe at the humeral area; the second near the vertical through the pelvic‐fin origin; the third at the vertical through the anal‐fin origin; and the third at the lower portion of the caudal peduncle tip; in some specimens, a variably marked, usually diffuse pigmentation can be present in the midline of the flanks, between the pectoral and pelvic fins. In *P. vittata*, the most widespread pattern includes a dark blotch between the pectoral‐ and pelvic‐fin origins and a blotch near the vertical through of the anal‐fin base, and in male specimens, a small, round mark can be seen near the humeral area, as well as a vertically elongate blotch at distal tip of caudal peduncle. In *P. punctata*, the longitudinal series of blotches is more variable, ranging from 7 to 16 (see colouration in alcohol), as the blotches usually extend along the area of a single scale, contrarily of those in *P. spilota* and *P. vittata*, in which the blotches usually extend through two to three scales.

In addition to the aforementioned morphological and pigmentation characters allowing the distinction of *P. punctata*, the genetic data and the delimitation algorithms employed herein allowed the unambiguous distinction of the species from its most similar relative, *P. spilota*. In the Bayesian tree (Figure 4), the genetic distance between both species was 5.40%, a considerably high distance compared to the threshold usually employed for species delimitation. The use of DNA barcoding for the identification of Neotropical ichthyofauna is well established and efficient in species detection (see e.g., Nascimento et al., 2023; Santana et al., 2023; Silva‐Santos et al., 2023; Souza et al., 2023). This is especially relevant for taxonomically challenging groups such as *Pyrrhulina*, in which molecular approaches have recently indicated significant cryptic diversity (Souza et al., 2023), highlighting the need for integrative taxonomic studies (Roxo et al., 2015; Souza et al., 2018; Ferreira et al., 2022) to better understand and provide accurate diversity estimates for the group.

The examination of global biodiversity information facility distribution sites for *P. spilota* revealed likely mistaken identification problems with specimens originating from the Beni and Mamoré rivers (Rio Madeira system), in Bolivia, as the species is not expected to occur there. Instead, *P. vittata* is common and abundant in that basin (Netto‐Ferreira & Marinho 2013) and would correspond to a more accurate identification of that material. As previously stated, the highly similar pattern observed in both species has likely led to the confusion between both species, which is corrected herein.

**FIGURE 5:**
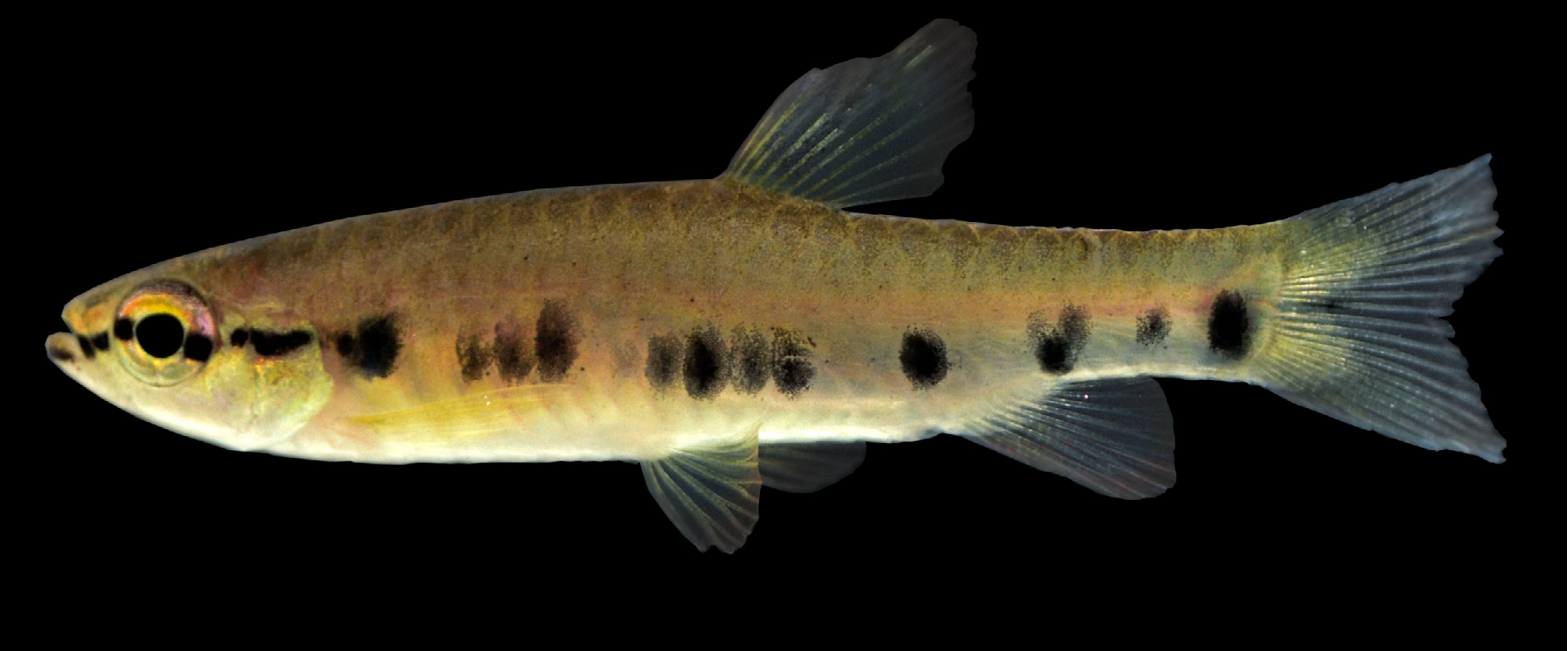
Uncatalogued aquarium specimen of *Pyrrhulina punctata*. Photo by Leonardo Davila.

Even though the horizontal blotches of large‐sized male specimens above 40.0 mm SL may coalesce, forming a more irregular pigmentation on the flanks, the condition observed in *P. punctata* can be easily distinguished from that of *P. lugubris* and *P. obermulleri*. Representatives of *P. punctate* lack clear areas near the scale focus on the flanks typical of those species, ranging from yellow to red in life, and present well‐marked black spots on the anal fin, as shown in Figure 5, whereas large males of the aforementioned species exhibit distinct pigmentation restricted to the distal border of the anal fin. Regarding female, juvenile and smaller‐sized males of *P. lugubris* and *P. obermulleri*, those can be promptly distinguished from the new species as they lack well‐marked pigmentation along the flanks.

### 3.7 Comparative material

In addition to the specimens listed in Netto‐Ferreira et al. (2011), Netto‐Ferreira (2012), Netto‐Ferreira and Marinho (2013), Netto‐Ferreira et al. (2013) and Vieira and Netto‐Ferreira (2019), the following specimens were examined. *P. spilota*. SU 52352 holotype (1, 52.5 mm SL), SU 52353 paratype (1, 44.3 mm SL), USNM 197523 paratypes (17, 52.1–23.3 mm SL), USNM 310999 (8, 35.4–44.6 mm SL), ZMB 32559 (11 of 12, 48.0–55.12 mm SL).

## AUTHOR CONTRIBUTIONS

All authors contributed to the theoretical framework and writing of the manuscript. Taina Souza performed the molecular analyses. James Garcia‐Ayala produced the figures and distribution map. Lorena Vieira led the editing and revision of the manuscript. All authors reviewed the final manuscript.

## FUNDING INFORMATION

Authors were financially supported by the Conselho Nacional de Desenvolvimento Científico e Tecnológico (CNPq) (313834/2021‐0 Produtividade em Pesquisa – Andre Netto‐Ferreira; 140177/2020‐5 PhD fellowship – Lorena Vieira), FAPERGS (72550.751.48979 Andre Netto‐Ferreira), Coordenação de Aperfeiçoamento de Pessoal de Nível Superior (CAPES) (Finance Code 001 to TBS – Taina Souza).
